# 1802. *Off-label* Use of Antimicrobial Agents at the University Hospital for Infectious Diseases, Zagreb, Croatia

**DOI:** 10.1093/ofid/ofac492.1432

**Published:** 2022-12-15

**Authors:** Iva Matković, Marija Santini

**Affiliations:** University Hospital for Infectious Diseases "Dr. Fran Mihaljević", Zagreb, Grad Zagreb, Croatia; School of Medicine, University of Zagreb; University Hospital for Infectious Diseases "Dr. Fran Mihaljević", Zagreb, Grad Zagreb, Croatia

## Abstract

**Background:**

Antimicrobial agents' (AMAs) o*ff-label* use can be associated with the prescriber's education. Little is known about the AMAs' *off-label* use by infectious disease (ID) specialists. We have conducted research at the University Hospital for Infectious Diseases (UHID), Zagreb, Croatia, a 230-bed tertiary center where ID specialists primarily prescribe AMAs.

**Methods:**

A retrospective cross-sectional analysis was conducted on the day with the highest hospitalization number, January 17, 2019. Any use of AMA not complying with the SmPC was considered o*ff-label* and evaluated regarding modality (indication, dose, regimen, route of administration, and age) and justification by institutional or/and the European Society of Clinical Microbiology and Infectious Diseases (ESCMID) guidelines.

**Results:**

The study included 155 patients, 63 (40.6%) adults and 92 (59.4%) children. 61 (96.8%) adult patients had at least one AMA. There were 134 AMA prescriptions in adults, 42 (31.3%) *off-label*. AMAs were most frequently prescribed *off-label* regarding regimen (21), dose (17), and indication (14). Institutional guidelines justified *off-label* in 5 cases regarding dose, 4 regarding indication, and 2 regarding regimen. ESCMID guidelines justified o*ff-label* prescriptions in 5 cases regarding indication. The rest of the *off-label* was not supported by the existing guidelines. 60 (65.2%) pediatric patients had at least one AMA. There were 73 prescriptions, 31 (42.5%) *off-label*. AMAs were most frequently prescribed *off-label r*egarding indication (19), dose (12), and age (2). Institutional guidelines justified 3 *off-label* cases regarding indication and 1 regarding dose. ESCMID guidelines justified no *off-label* in children. The lack of guidelines was the leading cause of the *off-label* AMAs use in children.

**Conclusion:**

One-third of AMAs are prescribed *off-label* regarding SmPC by ID specialists. However, institutional and international guidelines can justify part of this use.

**Disclosures:**

**All Authors**: No reported disclosures.

